# The interrelationship between metabolic memory, glycemic variability, oxidative stress and microalbuminuria in type 1 diabetes

**DOI:** 10.1186/1758-5996-7-S1-A27

**Published:** 2015-11-11

**Authors:** Tatiana Valente, Fernando Valente, Maria Beatriz Bastos Lucchesi, Celia Soares Bittencourt, Monica Andrade Lima Gabbay, Sergio Atala Dib

**Affiliations:** 1 UNIFESP, São Paulo, Brazil

## Background

Several studies have discussed the role of metabolic memory (MM) and glycemic variability (GV) in the development of chronic diabetes complications (CDC), possibly by triggering oxidative stress (OS). However, the Results are conflicting and there are few studies in type 1 diabetes (T1D) patients.

## Aims

It was to investigate the relationship between metabolic memory, glycemic variability, oxidative stress and microalbuminuria (MA) in T1D.

## Patients and materials and methods

Seventh-six T1D without clinical CDC and 22 healthy individuals were studied. MM was evaluated by glycated hemoglobin (HbA1c) of the last 3 yrs. GV: “short term” (STGV) by standard deviation (SD) of continuous glucose monitoring system (CGMS) over 3 consecutive days and “long term” (LTGV) by SD of the last 3 months on Accu Chek 360® diabetes management system. OS biomarkers (OSB) were estimated from 8 h overnight urinary excretion rates of 8-isoprostaglandin-F2α (ELISA, ALPCO-US), from plasma nitric oxide (NO) by chemiluminescence; plasma thiobarbituric acid reactive substances (TBARS) and erythrocytes reduced/oxidized glutathione (GSH/GSSH) by colorimetric assay (EnzyChrom GSH/GSSH Assay- EGTT-100). HbA1c (HPLC; nv: 4.0-5.5%) and MA (imunoturbidimetric assay; nv <15 µg/min).

## Results

T1D (age: 23.6±6.8 yrs, disease duration: 13.0±6.0 yrs., BMI: 23.8±3.6 Kg/m2 and HbA1c (mean±SD) of the last 3 yrs.: 8.9±1.5%) and controls (age: 25.8±3.9 yrs, HbA1c: 5.4±0.3% and BMI: 22.1±2.8 Kg/m2 were studied. STGV was higher in T1D than in controls (74.5±19.7 vs 10.8±1.7 mg/dL; p< 0.001) and had association with LTGV (rS: 0.36; p: 0.003), age (rS: -0.23; p: 0.037) and HbA1c (p: 0.001). LTGV (103±26.1 mg/dL) in T1D was associated with BMI (rS: 0.24; p: 0.047). NO was higher in T1D than controls: 115±104.1 vs 63.8±13.6 μM (p: 0.001) and in T1D was correlated with LTGV ( rS: 0.28 ; p: 0.036), MA (rS: 0.26; p: 0.049) and the HbA1c (mean of the last year; rS: 0.27; p: 0.042). TBARS was correlated to MA (rS: 0.32; p: 0.015). The GSH/GSSH and the 8-iso-PGF-2α didn't show any correlation with the parameters studied.

## Conclusion

The OSB have a heterogeneous behavior in T1D. NO is higher in young adult with T1D and it was related with MM, LTGV and endothelial dysfunction. So these variables should be included on the objectives of T1D good glycemic control.

